# Magnetic resonance imaging of pelvic floor dysfunction - joint recommendations of the ESUR and ESGAR Pelvic Floor Working Group

**DOI:** 10.1007/s00330-016-4471-7

**Published:** 2016-08-03

**Authors:** Rania Farouk El Sayed, Celine D. Alt, Francesca Maccioni, Matthias Meissnitzer, Gabriele Masselli, Lucia Manganaro, Valeria Vinci, Dominik Weishaupt, On behalf of the ESUR and ESGAR Pelvic Floor Working Group

**Affiliations:** 1 Genitourinary & Pelvic Floor MR Imaging Unit, Department of Radiology, Faculty of Medicine, Cairo University Hospitals, Cairo, Egypt; 2 Department of Diagnostic and Interventional Radiology, University Dusseldorf, Medical Faculty, Duesseldorf, Germany; 3 Department of Radiological Sciences, Oncology and Pathology Policlinico Umberto I Hospital, Sapienza University Rome, Viale Regina Elena, Rome, Italy; 4 Department of Radiology, University Hospital Salzburg, Paracelsus Medical University, Salzburg, Austria; 5 Department of Radiology Dea, Umberto I Hospital, Sapienza University of Rome, Viale del Policlinico, Rome, Italy; 6 Department of Radiological Sciences, Policlinico Umberto I Hospital, Sapienza University of Rome, Viale Regina Elena, Rome, Italy; 7 Institute of Radiology and Nuclear Medicine, Triemli Hospital Zurich, Zurich, Switzerland

**Keywords:** MRI pelvic floor, MR defecography, Recommendations, ESUR, ESGAR

## Abstract

**Objective:**

To develop recommendations that can be used as guidance for standardized approach regarding indications, patient preparation, sequences acquisition, interpretation and reporting of magnetic resonance imaging (MRI) for diagnosis and grading of pelvic floor dysfunction (PFD).

**Methods:**

The technique included critical literature between 1993 and 2013 and expert consensus about MRI protocols by the pelvic floor-imaging working group of the European Society of Urogenital Radiology (ESUR) and the European Society of Gastrointestinal and Abdominal Radiology (ESGAR) from one Egyptian and seven European institutions. Data collection and analysis were achieved in 5 consecutive steps. Eighty-two items were scored to be eligible for further analysis and scaling. Agreement of at least 80 % was defined as consensus finding.

**Results:**

Consensus was reached for 88 % of 82 items. Recommended reporting template should include two main sections for measurements and grading. The pubococcygeal line (PCL) is recommended as the reference line to measure pelvic organ prolapse. The recommended grading scheme is the “Rule of three” for Pelvic Organ Prolapse (POP), while a rectocele and ARJ descent each has its specific grading system.

**Conclusion:**

This literature review and expert consensus recommendations can be used as guidance for MR imaging and reporting of PFD.

***Key points*:**

*• These recommendations highlight the most important prerequisites to obtain a diagnostic PFD-MRI.*

*• Static, dynamic and evacuation sequences should be generally performed for PFD evaluation.*

*• The recommendations were constructed through consensus among 13 radiologists from 8 institutions.*

## Introduction

Imaging of the female pelvic floor is of rising interest due to an ageing population, harboring an increasing incidence of pelvic floor disorders (PFD) and the rising need for comprehensive diagnosis and treatment. The Population Reference Bureau reported the percentage of the population aged 65 and older to be 13 % of the total population in the U.S. in 2010 with an expected increase to 20 % in 2050, whereas in Europe, the percentage was reported around 18 % in 2010 with an expected increase to 28 % in 2050 [1]. Women that are affected by PFD, often complain most about the impairment of their quality of life and ask for sufficient therapy, which is commonly surgical repair [2, 3]. Thus, imaging techniques have been constantly developed in recent years to support therapy planning and management. Magnetic resonance imaging (MRI) of the female pelvic floor, particularly, combines high-resolution images with an excellent soft tissue contrast and provides the possibility to assess noninvasively and more objectively a spectrum of possible disorders affecting the pelvic floor in one examination [4–7]. There is general agreement that MRI of the pelvic floor should encompass static and dynamic MR images, whereas dynamic means imaging under maximum stress to the pelvic floor and MR defecography. Static MR images visualize pelvic floor anatomy and defects of the supporting structures, while dynamic MR images visualize pelvic organ mobility, pelvic floor weakness, pelvic organ prolapse (POP) and associated compartment defects [5, 8–11]. Additionally, MRI may diagnose unexpected underlying masked functional abnormalities, which might be discrepant from the dominant symptom and may influence the choice of the surgical technique in around 42 % of patients with different spectra of PFD [12, 13].

Several studies and detailed reviews are published about MRI of the pelvic floor and different acronyms have been used for this examination including static and dynamic MR of the pelvic floor, MR defecography or MR proctography [4, 12, 14–16]. However, to date, there is neither consensus on a standardized imaging protocol nor on a systematic reporting scheme for MR-imaging of PFD. This may be due to the complexity of the anatomy and the functional interaction of the organs with the supporting structures resulting in a broad spectrum of PFD. Another important factor that contributes to this lack of consensus is the fact that PFD is treated by urologists, urogynecologists or proctologists. Consequently, each clinician may manage the patients’ condition from a different perspective. Therefore, MR-imaging acquisition varies according to the referring specialty and their rudiments for proper management and treatment decision. The wide range of different available MR protocols and a lack of standardization additionally increase variation between different centers. There is, therefore a necessity for recommendations from an expert panel that clearly defines the minimum prerequisites to obtain a state-of-the-art MR examination of the pelvic floor. This paper reports the recommendations of a panel of expert radiologists in pelvic floor imaging, which are joined in the pelvic floor-working group, which is under the umbrella of the European Society of Urogenital Radiology (ESUR) and the European Society of Gastrointestinal and Abdominal Radiology (ESGAR).

## Materials and methods

**Fig. 1:**
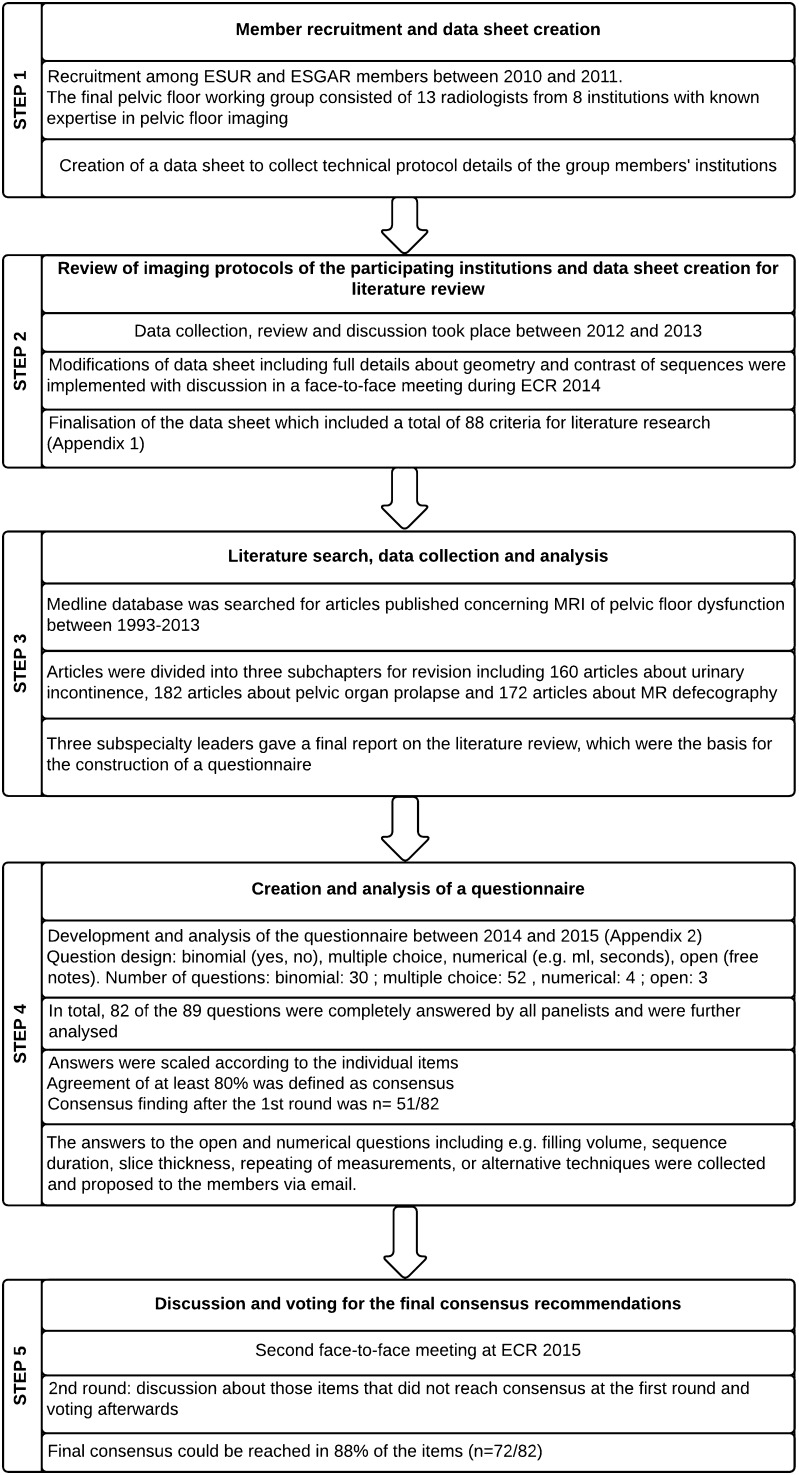
Flow chart of the five basic steps of the study

The study went through five basic steps that are displayed in Fig. 1.

Step 1 Member recruitment and data sheet creation

Participants for the working group were recruited among ESUR and ESGAR members between 2010 and 2011. The final working group consisted of 13 radiologists from one Egyptian and seven European institutions, all with known expertise in pelvic floor imaging. One member (RFE) created a data sheet to collect technical protocol details of the members’ institution. This sheet focused on information about the clinical referrer, patient population, patient preparation, and MR technique (hardware, imaging sequence and imaging parameters).

Step 2 Review of imaging protocols of the participating institutions and data sheet creation for literature review

Data collection, review and discussion of all imaging protocols of the participating institutions took place between 2012 and 2013. During this period modifications on the data sheet were implemented by (RFE) in which full details about both the geometry and the contrast of the static and dynamic MRI during straining as well as those of MRI defecography were added to the original data sheet. The results were presented and discussed in a face-to-face meeting during ECR 2014 during which a consensus was reached to finalize the data sheet for literature research (Appendix 1).

Step 3 Literature search, data collection and analysis

Literature search was conducted in the Medline database for articles published between 1993 and 2013 using the following keywords: “MRI AND Pelvic Floor“, “MRI defecography“, “MRI pelvic organ prolapse“, “MRI anal incontinence“, “MRI stress urinary incontinence“, “MRI AND defecography“, “Pelvic obstruction syndrome and MRI“, “Pelvic outlet obstruction and MRI“, “MRI and fecal incontinence“, “Pelvic floor and MRI“, “MRI and urinary incontinence“ and “Pelvic organ prolapse and MRI“.

Inclusion criteria were original data with full information about the parameters and the protocol of the examination that matched with our final data collection sheet for literature review.

Articles that were not written in English, did not deal with a human study population or lack of information about the performance of the examination were excluded.

The papers concerning MRI of PFD were divided by (RFE) into the following subchapters: urinary incontinence (160 articles), pelvic organ prolapse (182 articles) and MR-defecography (172 articles). Paper revision and data extraction was divided among participating members into three subspecialty groups (urology, gynaecology and proctology) with one leader for each group (GM, CDA, DW). Each subspecialty leader wrote a final report summarizing the data that was agreed upon. The collected evidence by this literature analysis was used to extract the relevant topics, which should be addressed by the working group panelists in order to construct a questionnaire.

Step 4 Creation and analysis of a questionnaire

From October 2014 to March 2015, one author (CDA) developed a questionnaire to define the most important information and requisites needed to perform MRI of PFD with standardized imaging protocol and reporting scheme. It was finalized in consensus with one author of ESGAR (DW). Since all panelists are using MR systems with a conventional closed-magnet design where the patient can only be examined in supine (lying) body position, procedural and technical aspects of pelvic floor imaging was focused to this type of magnet design. The questionnaire included binomial, multiple choice, numerical and open questions, in total 89 items (Appendix 2). This questionnaire was mailed to all panelists. In total, 82 of 89 questions were answered by all experts and were scaled according to the individual item in question for further analysis. The data obtained were analyzed using descriptive statistics. Agreement of at least 80 % was defined as consensus finding.

Step 5 Discussion and voting for the final consensus recommendations

The second face-to-face meeting took place during ECR congress in 2015. For those questions that did not reach consensus at the first round of the questionnaire analysis, wording was modified to obtain better-defined statements subjected for voting by the experts in a face-to-face meeting. During that meeting the panelists discussed those items and were asked to vote. However, there were items that did not reach consensus but were reported by number of panelist to be important and warrants being included in the recommendations. These items were re-analyzed, and those that were found to be supported by case control or cohort studies from the literature, in particular level of evidence 2 according to the sign criteria, whereas expert opinion is level of evidence 4 (www.sign.ac.uk), were also included in the final recommendation.

## Results

Consensus was reached for 88 % of 82 items and the recommendations regarding indication, patient preparation, imaging protocol, criteria for MRI assessment and reporting were constructed from these.

### Indications for MR imaging of pelvic floor dysfunction

The indications for MR imaging of the pelvic floor that scored the highest number of agreement among the group members and the literature review are rectal outlet obstruction (92 % agreed upon), rectocele (92 % agreed upon), recurrent pelvic organ prolapse (POP) (85 % agreed upon), enterocele (85 % agreed upon) and dyssynergic defecation (anismus)(85 % agreed upon) (Table 1).

**Table 1:** Most common indications for MR-imaging of pelvic floor dysfunction*

Indications	Score of agreement achieved**
Anterior compartment	
Stress urinary incontinence	7/13
Recurrence after surgical POP repair	7/13
Middle compartment	
Recurrence after surgical POP repair	11/13
Enterocele / Peritoneocele	11/13
POP	7/13
Posterior compartment	
Outlet obstruction	12/13
Rectocele	12/13
Anismus	11/13
Fecal incontinence	10/13
Recurrence after surgical POP repair	9/13
Rectal intussusception	8/13
Non-specific compartment	
Pelvic pain / perineal pain	7/13
Descending perineal syndrome	7/13

*POP* pelvic organ prolapse

* The indications of MRI in each compartment are listed in a descending order from those that scored the highest number of agreement among both the group members and the literature review

** Number of group members *n* = 13

### Patients’ preparation and hardware requirements

Full patients’ history of pelvic floor disorder should be taken prior to scanning (consensus 100 %). The patient should be examined at least in a 1.5 T MRI unit with a phased array coil, as this is the most agreed-upon field strength (consensus 100 %). The patient is examined in the supine position with the knees elevated (e.g. on a pillow with firm consistency) as this was found to facilitate straining and evacuation (consensus 100 %). The coil should be centered low on the pelvis to ensure complete visualization of prolapsed organs [4, 15]. The bladder should be moderately filled, therefore voiding 2 hours before the examination is recommended (consensus 100 %).

Prior to the examination the patient should be trained on how to correctly perform the dynamic phases of the examination and the evacuation phase (consensus 100 %). The patient is instructed to squeeze as if trying to prevent the escape of urine or feces and hold this position for the duration of the sequence. For maximum straining, the patient is instructed to bear down as much as she/he could, as though she/he is constipated and is trying to defecate [15]. For the evacuation phase, the patient should be instructed to repeat the evacuation process until the rectum is emptied.

To decrease possible patient’s discomfort, a protective pad or a diaper pant should be offered to the patient, which helps to increase patients’ compliance during dynamic and evacuation phases (consensus 100 %). No oral or intravenous contrast is necessary [15].

The rectum should be distended in order to visualize the anorectal junction (ARJ), rectoceles and intussusceptions, and to evaluate the efficacy of rectal evacuation (consensus 100 %). Ultrasound gel is the recommended medium to distend the rectum, however, the amount varies between 120 to 250 cc (consensus 100 %). For rectal distension a large amount of gel (180-200 cc) likely improves the capacity of the patient to defecate. A checklist for the recommended patients’ preparation is listed in (Table 2).

A rectal cleansing enema prior to the examination is helpful but reached no consensus to be generally performed. Vaginal filling with 20 cc ultrasound gel is helpful for better demarcation, however, it reached no consensus for general performance and its application may be limited due to social or religious backgrounds.

**Table 2:**
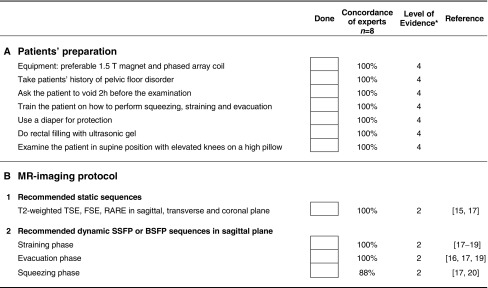
Checklist for the recommended patients’ preparation and MR-Imaging protocols

*BSFP* balanced state free precession, *FSE* fast spin echo, *RARE* rapid acquisition with relaxation enhancement, *SSFP* steady state free precession*, TSE* turbo spin echo

* Level of evidence 2 = based on systematic reviews, case control or cohort studies; Level of evidence 4 = based on expert opinion (www.sign.ac.uk)

### MR-imaging protocol

The recommended MR-imaging protocol is summarized in (Table 3). The protocols consists of static MR sequences and dynamic sequences, whereas dynamic means imaging during straining, squeezing and during evacuation or defecation.

**Table 3:** Recommended MR-imaging protocols

Plane		Sequence	Technique	TE (ms)	TR (ms)	ST (mm)	FOV (mm)	Matrix	Angulation	Number of slices	Level of evidence*
Static MRI sequences 2D MRI											
	Sagittal	T2WI	Turbo/fast spin echo	77-132	500-4210	4	200-300	256-448	Midsagittal	23	2
	Transverse	T2WI	Turbo/fast spin echo	88-132	500-7265	4	200-300	256-512	Perpendicular to the urethra	25	2
	Coronal	T2WI	Turbo/fast spin echo	80-132	500-7265	4	200-260	256-512	parallel to the urethra	26	2
Dynamic MR sequences											
Squeezing											
	Sagittal	T2WI	GE, FFE	1.27-1.88	3.3-397.4	8	250-310	126-280	Midsagittal	1 or 3	2
Straining											
	Sagittal	T2WI	GE, FFE	1.27-1.88	3.3-397.4	8	250-310	126-280	Midsagittal	1 or 3	2
*optional* ^*a*^	Transverse	T2WI	GE, FFE	1.6-80	5.0-1200	5 or 6	250-310	126-280	Perpendicular to the urethra	5	2
*optional* ^*b*^	Coronal	T2WI	GE, FFE	1.6	5	5 or 6	300	256	Parallel to the urethra	5	2
MR-Defecography											
	sagittal	T2w	GE, FFE	1.27-1.88	3.3-397.4	8	250-310	168-280	Midsagittal	1 or 3	2
*optional* ^*c*^	coronal	T2w	GE, FFE	1.27-1.6	5-397	4 or 8	257-350	154-256	Parallel to anorectum	5	2

*FFE* fast field echo, *FOV* field of view*, GE* gradient echo, *ST* slice thickness, *2D* two-dimensional, *TE* time of echo*, TR* time of repetition, *T2WI* T2-weighted

^a^Technique was reported by 3/8 experts and is supported by reference [15, 21]

^b^Technique was reported by 3/8 experts and is supported by reference [15, 21]

^c^Technique was reported by 3/8 experts and is supported by reference [22]

* Level of evidence 2 = based on systematic reviews, case control or cohort studies; Level of evidence 4 = based on expert opinion (www.sign.ac.uk)

**Fig. 2:**
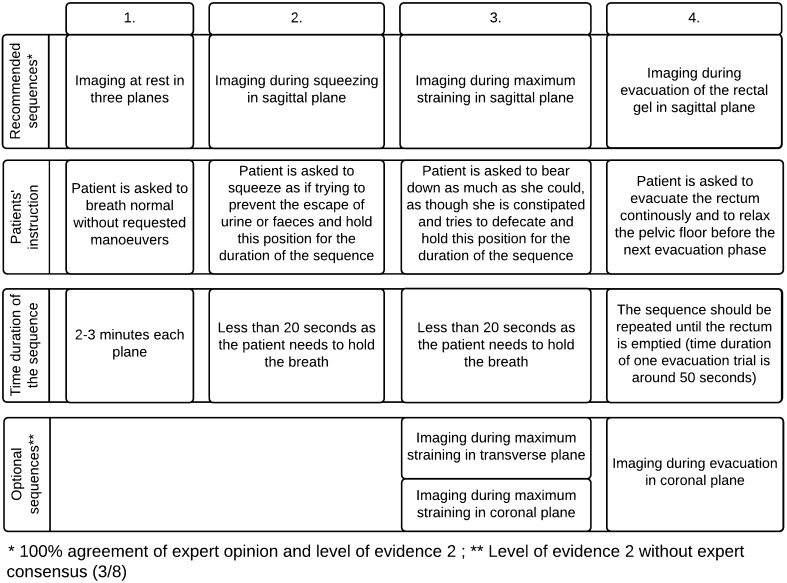
Schedule of the recommended imaging sequences, the instruction given to the patient and the time duration per sequence

According to the concordance of experts and level of evidence, high resolution T2-weighted images (T2WI) (e.g. Turbo Spin Echo, TSE ; Fast Spin Echo, FSE; Rapid Acquisition with Relaxation Enhancement, RARE) in three planes are recommended for static images, whereas steady state (e.g. FISP, GRASS, FFE, PSIF, SSFP, T2-FFE) or balanced state free precession sequence (e.g. trueFISP, FIESTA, B-FFE) in sagittal plane is recommended for dynamic sequences (squeezing and straining) and evacuation sequence (consensus 100 %). The dynamic sequence should not exceed 20 seconds each, as breath holding is required (consensus 100 %). The evacuation sequence should be repeated until the rectum is emptied to exclude rectal intussusception (total time duration around 2-3 minutes)(consensus 100 %). Dynamic MR imaging during evacuation is mandatory, because certain abnormalities and the full extent of POP is only visible during evacuation. Optional MRI sequences can be added and acquired for further assessment of pelvic floor relaxation. These include axial and coronal dynamic sequences during maximum straining. Illustration of all the recommended imaging sequences and patients’ maneuvers is summarized in (Fig. 2).

Since the performance of adequate pelvic stress during the dynamic sequences is important in order to assess the full extent of PFD, quality control of the study is essential. The study can only be considered diagnostic if a clear movement of the abdominal wall is seen during squeezing and straining. If no evacuation of rectal content at all or a delayed evacuation time (more than 30 seconds to evacuate 2/3 of the rectal content) is present, anismus should be considered (consensus 88 %) [23].

### Image analysis, measurements, grading and MRI report

#### Image analysis

**Fig. 3:**
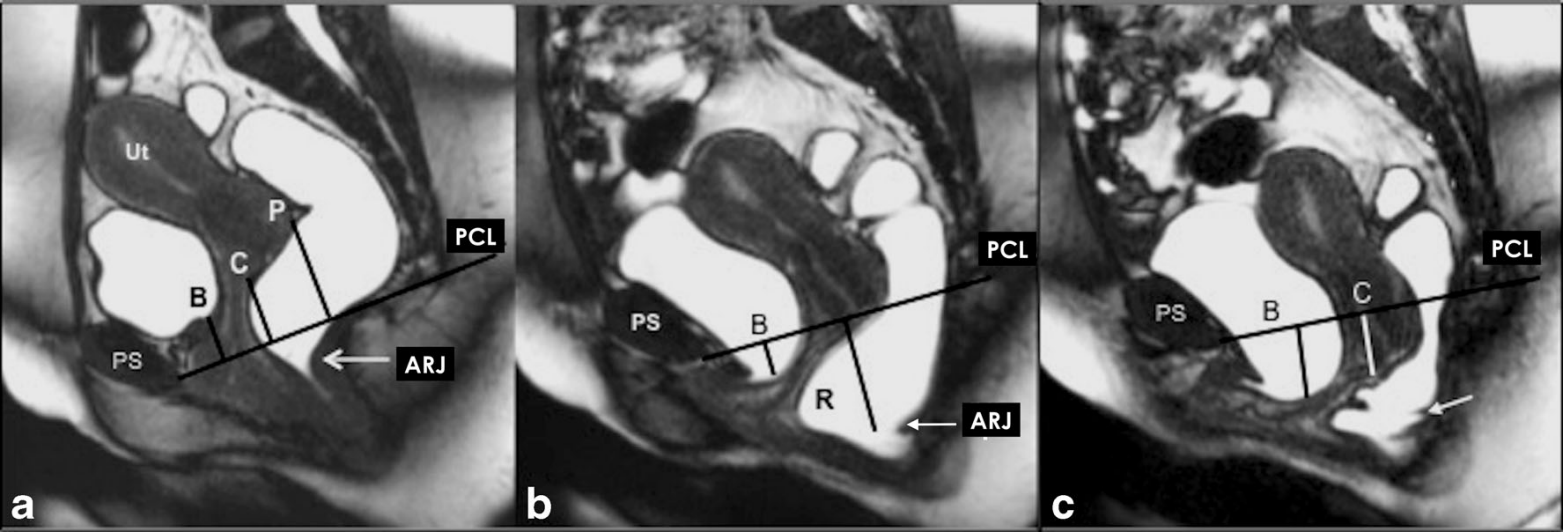
Basic measurements. **a**. Dynamic Balanced Fast Field Echo (BFFE) sequence in the midsagittal plane at rest shows how to plot the basic measurements of pelvic organ prolapse. The pubococcygeal line (PCL), drawn on sagittal plane from the inferior aspect of the pubic symphysis (PS) to the last coccygeal joint. After defining the PCL, the distance from each reference point is measured perpendicularly to the PCL at rest and at maximum straining. B; bladder base, C; cervix, P; pouch of Douglas, ARJ; Anorectal junction. Measured values above the reference line have a *minus sign*, values below a *plus sign*. **b**. Dynamic BFFE during maximum straining shows the movement of the organs compared to their location at rest. It is recommend to give the difference of the values at rest and during straining for each organ-specific reference point (pelvic organ mobility). R; Rectocele, ARJ; Ano-Rectal Junction. **c**. MRI defecography (BFFE) in the mid sagittal plane during evacuation of the intra-rectal gel. Dynamic MR imaging during evacuation is mandatory, because certain abnormalities and the full extent of POP are only visible during evacuation. In this case compared to the maximum staining phase it is obvious that there is increase of the degree of the pelvic organ descent and development of new pathology including the loss of urine and the detection of masked intussusception, which was detected only during excavation (white *arrow*)

A clear consensus was reached that the assessment of a MR study of the pelvic floor should include analysis of static images for detection and classification of structural abnormalities. The dynamic images are analyzed with regard to functional abnormalities that are assessed by metric measurements of the three compartments of the pelvic floor (consensus 100 %) (Fig. 3). The measurements help to recognize and grade the extent of POP and pelvic floor relaxation (PFR), as well as they are used to grade anterior rectoceles and enteroceles (consensus 100 %). Both static and dynamic MRI findings as well as the results of the metric measurements should be reported in a structured MR reporting scheme (consensus 100 %) (Table 4).

**Table 4:**
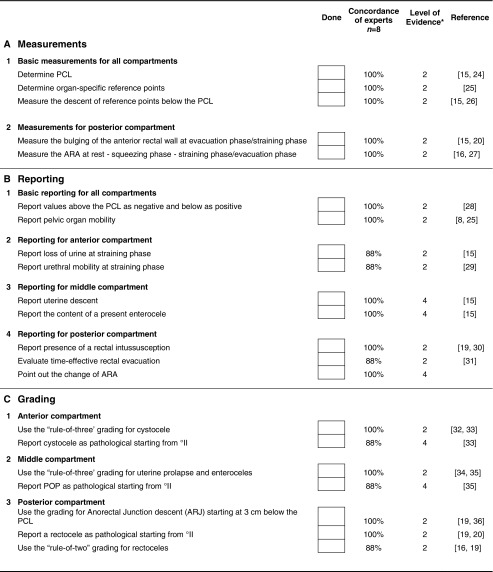
Checklist for the recommended MRI reporting scheme

*PCL* pubococcygeal line, *ARA* anorectal angle, *POP* pelvic organ prolapse, *ARJ* anorectal junction

* Level of evidence 2 = based on systematic reviews, case control or cohort studies; Level of evidence 4 = based on expert opinion (www.sign.ac.uk)

Due to the different views of the clinical specialists involved in the treatment of PFD, it is suggested to consider adapting the MRI reporting scheme according to the specialty of the referring physician. A proposal for a specialty-based MRI report is given in (Table 5).

**Table 5:** Specialty-based MRI reporting scheme

Urologic patients
Report of pathologies if present
During dynamic sequences
Loss of urine through the urethra at maximum straining
Hypermobility of the urethra
Kinking of the vesicourethral junction
Uretherocele
Cystocele; type (distension or displacement), size (cm), grade
On static images
Damage of the supporting urethral ligaments
Avulsion or defect of the puborectal muscle
Measurements
Pelvic organ mobility
Pelvic floor relaxation
Iliococcygeus angle
Hiatal dimensions
Further evaluation
Additional findings regarding the pelvic organs*
Coexistent middle and posterior compartment disorders
(Uro)gynecologic patients
Report of pathologies, if present:
During dynamic sequences
Cystocele; type (distension or displacement), size (cm), grade
Uterine prolapse: partial or total
Enterocele: type (content of the peritoneal sac), size (cm), grade
On static images
Avulsion or defect of the puborectal muscle
Measurements
Pelvic organ mobility
Pelvic floor relaxation
Iliococcygeus angle
Hiatal dimensions
Further evaluation
Additional findings regarding the pelvic organs*
Coexistent anterior and posterior compartment disorders
Proctologic patients
Report of pathologies, if present:
During dynamic sequences
Rectocele: type (anterior or rarely posterior) size (cm), grade
Rectal mucosal invagination or prolapse: differentiation, extent, grade
Rectal descent: distance to PCL (cm), grade
Enterocele: type (content of the peritoneal sac), size (cm), grade
Lack of changes of ARA
Insufficient opening of the anal canal with inadequate rectal emptying during evacuation
Rectal intussusception
Measurements
Rectocele
Rectal decent
ARA
Pelvic organ mobility
Pelvic floor relaxation
Further evaluation
Additional findings regarding the pelvic organs*
Coexistent anterior and middle compartment disorders

*ARA* anorectal angle, *PCL* pubococcygeal line, *PFD* pelvic floor disorder.

* e.g. adnexal lesions, uterine diseases, urethral and bladder diverticula, diverticulosis, diverticulitis

#### Measurements

The pubococcygeal line (PCL), drawn on sagittal plane from the inferior aspect of the pubic symphysis to the last coccygeal joint, is recommended as reference line to measure POP (consensus 100 %). It shows the highest inter- and intraobserver reliability of MRI measurements in women with POP of the anterior and middle compartment compared to all proposed reference lines in the literature with an intercorrelation coefficient (ICC) between 0.70-0.99 (Fig. 3a) [14, 37, 38].

After defining the PCL, the distance from each reference point is measured perpendicularly to the PCL at rest and at maximum strain (consensus 100 %) [26, 29]. In the anterior compartment, the organ-specific reference point is the most inferior aspect of the bladder base (B), in the middle compartment, the organ-specific reference point is the anterior cervical lip (most distal edge of the cervix)(C), or the vaginal vault in case of previous hysterectomy (V), and in the posterior compartment, the organ-specific reference point is the anorectal junction (ARJ) (consensus 100 %) (Fig. 3a) [15, 16, 20, 25, 29, 39]. Measured values above the reference line have a minus sign, values below a plus sign (consensus 100 %) [25].

Reporting of the movement of the organs compared to their location at rest is stated to give more valuable information for the referrer than a grading system alone [8, 25]. We therefore recommend giving the difference of the values at rest and during straining for each organ-specific reference point (pelvic organ mobility)(consensus 100 %) (Fig 3a, b).

**Fig. 4:**
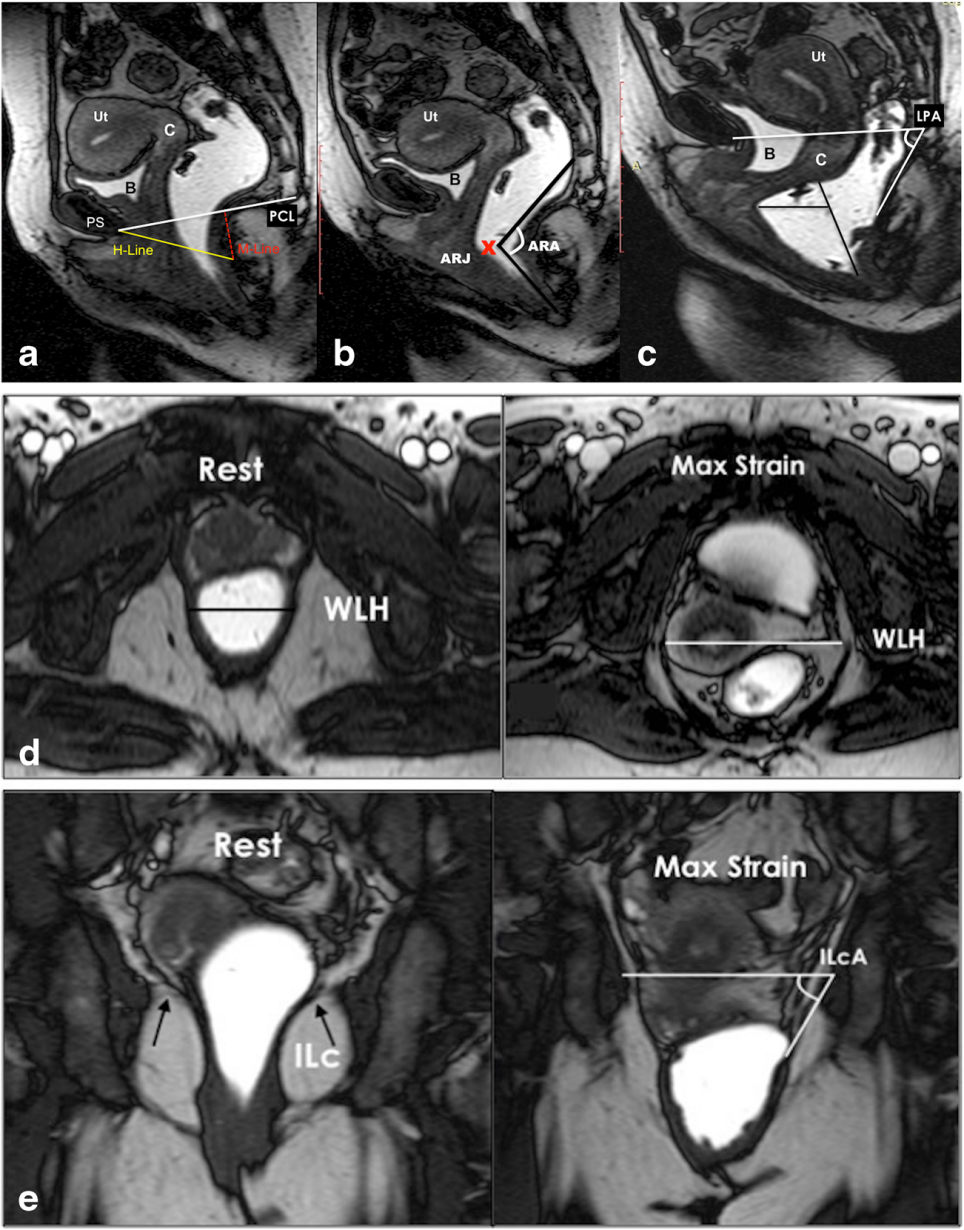
Pelvic floor relaxation and posterior compartment measurements. **a**,**b**,**c** Dynamic Balanced Fast Field Echo (BFFE) sequence in the midsagittal plane at rest (**a**) , mild (**b**), and maximum straining (**c**). (**a**) shows how to quantify the pelvic floor laxity. The H-line extends from the inferior aspect of the pubic symphysis to the anorectal junction, the M-line is dropped as a perpendicular line from the pubococcygeal line (PCL) to the posterior aspect of the H-line. (**b**) Demonstrates the anorectal angle (ARA) drawn along the posterior border of the rectum and a line along the central axis of the anal canal on sagittal plane. ARJ; Ano-Rectal Junction. (**c**) Shows how to measure and diagnose a pathological rectocele: a line drawn through the anterior wall of the anal canal is extended upward, and a rectal bulge of greater than 2 cm anterior to this line is described as a rectocele (R). The levator plate angle (LPA) is enclosed between the levator plate and the PCL. **d**,**e**. Dynamic Balanced Fast Field Echo (BFFE) sequence in axial (**d**) and coronal (**e**) plane at rest and during maximum straining. In the axial plane the width of the levator hiatus is enclosed between the puborectalis muscle slings. On the coronal plane, the iliococcygeus angle is measured between the iliococcygeus muscle and the transverse plane of the pelvis in posterior coronal images at the level of the anal canal

A rectocele is diagnosed as an anterior rectal wall bulge and it is measured during maximum straining and evacuation (Fig 4). Typically, a line drawn through the anterior wall of the anal canal is extended upward, and a rectal bulge of greater than 2 cm anterior to this line is described as a rectocele (consensus 100 %) [28, 34]. The anorectal angle (ARA) should be drawn along the posterior border of the rectum and a line along the central axis of the anal canal on sagittal plane (Fig. 4b) at rest, squeezing and maximum straining (consensus 100 %) [20, 27].

Pelvic floor relaxation (PFR) often coexists with POP, but it is a different pathologic entity. For quantification of the weakness of the levator ani and to reflect pelvic floor laxity, five measurements can be performed [15] , however, it reached no consensus to measure it routinely. The length of the hiatus (H-line), the descent of the levator plate (M-line) and the levator plate angle are evaluated in the sagittal plane (Fig 4a, c), whereas the transverse width of the levator hiatus and the iliococcygeus angle are assessed in the axial and coronal plane during maximum straining(Fig. 4e,d) [15]. Table 6 provides an overview of the entire spectrum of the published reference values for quantitative MR-measurements of the pelvic floor.

**Table 6:** Overview of the published reference values for quantitative MR-measurements of the pelvic floor

Parameters	Reference value ± standard deviation	Reference
Anterior compartment		
Bladder base position (according to PCL) at rest	−2.3 ± 0.46 cm	[39]
Bladder base position (according to PCL) during straining	0.81 ± 1.11 cm	[39]
Middle compartment		
Anterior cervical lip position (according to PCL) at rest	4.31 ± 0.78 cm	[39]
Anterior cervical lip position (according to PCL) during straining	−0.79 ± 1.65 cm	[39]
Posterior compartment		
Anterior bulge of the rectal wall during straining (rectocele)	2.6 ± 0.6 cm	[39]
Ano rectal junction (ARJ) at rest	≤3 cm below the PCL 0.53 ± 0.99 cm	[34, 39]
ARJ during squeezing	Elevation of ARJ	[36]
ARJ during straining	2.99 ± 1.03 cm	[39]
Anorectal angle (ARA) at rest	85-95° 93° ± 4.8°	[31, 39]
ARA during squeezing	71° sharpening of 10-15°	[16, 27]
ARA during straining or defecation	103° 15-25° more obtuse 108° ± 14.7°	[16, 27, 39]
Measurements for quantification of the pelvic floor laxity		
H-line (hiatus) during straining	5.8 ± 0.5 cm	[15]
M-line (descent of H-line to PCL) during straining	1.3 ± 0.5 cm	[15]
Levator plate angle during straining	11.7 ± 4.8°	[15]
Iliococcygeus angle at rest	20.9 ± 3.5°	[15]
Iliococcygeus angle during straining	33.4 ± 8.2°	[15]
Transverse diameter of levator hiatus at rest	3.3 ± 0.4	[15]
Transverse diameter of levator hiatus during straining	4.5 ± 0.7 cm	[15]

#### Grading

The “Rule of three’ is the recommended grading system in the anterior and middle compartment starting at 1 cm below the PCL (Table 4) [15, 16, 32, 34, 40]. This is based on the fact that the pelvic floor may descend and widen up to 2 cm during abdominal pressure. Consequently, the pelvic organs follow the movement of the pelvic floor inferiorly but without protrusion through their respective hiatuses [4]. The bladder base, particularly, may descend up to 1 cm below the PCL during straining in continent women and should not be stated as a cystocele (consensus 100 %) [24, 34].

The “Rule of two” is recommended for grading the anterior rectal wall bulge in rectoceles (consensus 100 %) (Table 4) [16; 23; 25; 26; 31]. It should be reported as pathological from grade °II, as a grade °I rectocele can be observed in nearly 78-99 % of parous women, while rarely in men [20, 28, 41].

Anorectal junction descent (ARJD) is graded (grade °I) between 3 and 5 cm below the PCL, and (grade °II) with at least 5 cm (consensus 100 %) [36].

Small intussusceptions of the rectal wall are considered to be normal findings during defecation, observed in nearly 80 % of healthy subjects [41].

### Reporting other functional abnormalities and structural defects

#### Functional abnormalities on dynamic MR images

Loss of urine through the urethra during maximum straining records urinary incontinence (UI) and should be reported if present (consensus 88 %)[15]. Urethral hypermobility as a predictor for UI should be reported if present (consensus 88 %) [29]. If a cystocele is present, the differentiation of a distention or a displacement cystocele can be made, which is helpful for therapy planning, however it reached no consensus for general reporting [42].

If an enterocele is present, the report should include the content of the peritoneal sac, as clinical examination alone may have shortcomings in identifying the content (consensus 100 %) [5, 20, 22, 31, 43].

The end of evacuation phase is important to identify intussuception (Fig. 3c) [30].

The change of the ARA during dynamic and evacuation sequence compared to the ARA at rest expresses the functioning of the puborectal muscle. In particular, the ARA should sharpen during squeezing and should become more obtuse during straining and evacuation [16, 27, 39]. We recommend to report the individual function, as the literature presents with a widespread of normal reference values (consensus 100 %).

### Structural defects on static MR images

**Fig. 5:**
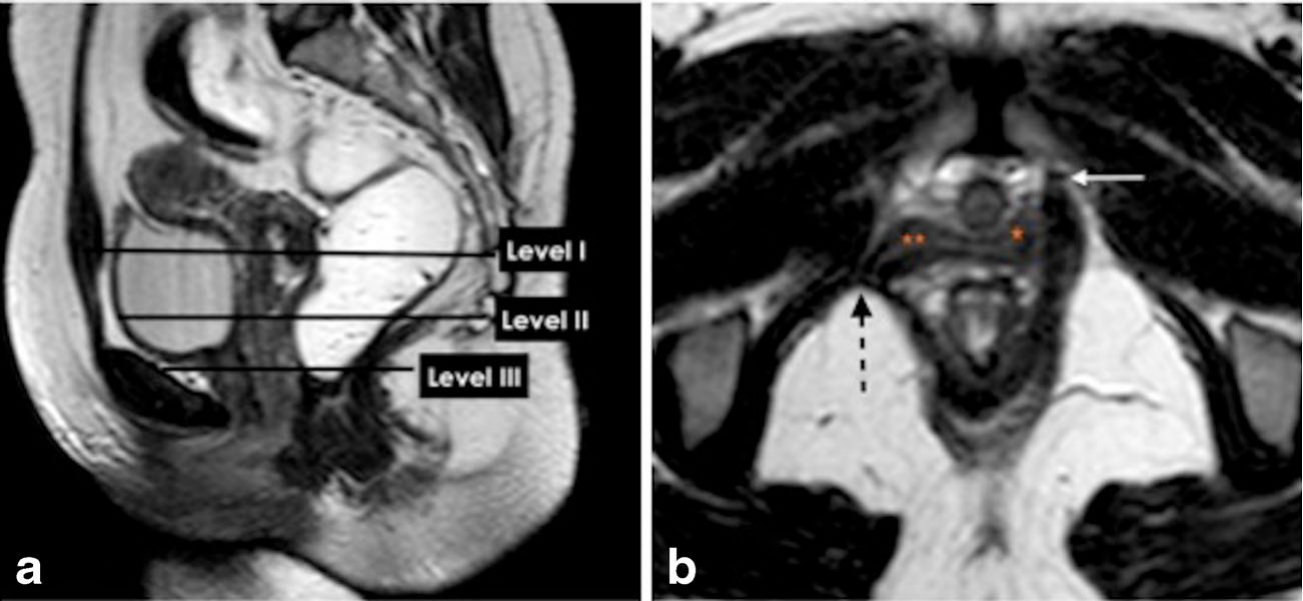
Functional three -part pelvic supporting system. **a**,**b**. Static T2W Turbo-Spin Echo (TSE) MR images in sagittal and axial plane. (**a**) Sagittal MR image illustrating the levels of the endopelvic fascia (paracolpium) that attaches the upper vagina to the pelvic walls, it is divided into three levels. Level I (suspension); the portion of the vagina adjacent to the cervix (the cephalic 2–3 cm of the vagina) functionally it provides the upper vaginal support. Level II (attachment); located in the mid portion of the vagina, it stretches the vagina transversely between bladder and rectum. The anterior vaginal wall provides urinary bladder support. The posterior vaginal wall and the endopelvic fascia (rectovaginal) form a restraining layer that prevents the rectum from protruding forward. (**b**) Axial T2W image shows detachment of the puborectalis muscle from its origin identified by discontinuity of its attachment to the pubic bone on the right side (*dotted black arrow*) (*white arrow*, normal bony attachment), (** loss of H-shaped vagina on the right side), (*; normal lateral vaginal attachment on the left side)

Description of structural defects and anatomical abnormalities, that are assessed in static T2WI are more likely specialty-based PFD-related questions from the referrer (Table 5). The functional three-part pelvic supporting system (Fig. 5) includes the urethral support system, which maintains urinary continence; the vaginal support system, which prevents prolapse; and the anal sphincter complex that maintains anal continence. Urethral support system defects may include urethral ligament defect and / or distortion, level III endopelvic fascial defects, or puborectalis muscle detachment(Fig. 5b), disruption, atrophy or avulsion [15, 18, 21, 33, 44–46]. The spectrum of vaginal support system abnormalities includes level I and II paravaginal fascial defects and/or iliococcygeus diffuse or focal muscle abnormality [35].

### Limitations of the study

The study has few limitations. Four panelists who participated in Step 1 and 2 of the study were from the same institution. Therefore, only 1 out of their 4 completed questionnaire was included in the final analysis to avoid biased results. Nevertheless, since all 8 panelists who have completed the questionnaire were from different institutions these recommendations can be considered to represent the entire spectrum of expert opinions in the field of pelvic floor MRI. Second, the recommendations given in this study with regard to technical aspects of MRI of the pelvic floor relate to conventional closed-configuration magnets for MR imaging allowing patient positioning in lying body position only. However, this is the most agreed upon scanner, in addition several studies have shown that patient positioning does not significantly influence diagnostic performance of MR imaging of the pelvic floor [17, 19, 47, 48].

## Conclusion

Based on an extensive literature review and analysis and of expert consensus, these proposed recommendations can be used as guidance for standardized MR imaging and reporting of PFD. Nevertheless, our joint ESUR-ESGAR pelvic floor-working group is aware about the complexity of the topic and that further studies are mandatory to achieve additional refinements of guidelines for MR imaging, diagnosing and reporting of PFD.

## Appendix 1 Data sheet created to collect the details of the technical protocols of the group members and for literature review

**Table:**

General				
Institution Name	Author	Referrer 1 = gynecologist 2 = urologist 3 = proctologist 4 = other	Indication for MRI of the pelvic floor	Compartment examined 1 = anterior 2 = middle 3 = posterior 4 = anterior and middle 5 = all of them

**Table:**

Patient preparation							
Preparation of upper GI-tract 0 = no 1 = yes	Rectal enema 0 = no preparation 1 = cleansing enema	Rectal filling 0 = no filling 1 = ultrasonic gel 2 = potato starch 3 = air	Volume of rectal filling (ml)	Use of urethral Folys catheter 0 = no 1 = yes	Bladder filling 0 = empty 1 = moderately filled 2 = full 3 = 1 h void 4 = 2 h void	Vaginal filling 0 = no filling 1 = sterile gel 2 = normal gel	Use of IV contrast 0 = no 1 = yes

**Table:**

Patient instruction and positioning		
Patient Training 0 = on grades of straining 1 = on evacuation 2 = on withholding	patient positioning 1 = supine 2 = sitting 3 = lateral right 4 = lateral left 5 = prone 6 = upright	patient positioning 0 = legs side by side 1 = legs separated 2 = knees elevated 3 = upright

**Table:**

MR scanner		
MR-scanner 1 = 1.0 T 2 = 1.5 T 3 = 3 T 4 = <1	MR-scanner 0 = conventional scanner 1 = open scanner 2 = upright scanner	Coil Selection

**Table:**

Imaging protocol		
Static MRI sequences	Dynamic cine MRI sequence during different patients' maneuvers Number of phases A = 3 phases (rest, squeezing, strain) B = 4 (rest, squeezing, moderate- max strain) C = 5 (rest, squeezing, mild- moderate -max strain)	MR Defecography 1 = real time fluoroscopy 2 = multiple repetitions

**Table:**

Geometry (for every sequence)							
Sequence 1 = T1w 2 = T2w	Plane 1 = tra 2 = sag 3 = cor	FOV (mm) RFOV(%) Fold over suppression	Matrix scan Matrix recon-struction Scan percentage	Number of slices Slice thickness (mm)	Slice gap Slice orientation	Fold over direction	REST slabs 1 = free 2 = parallel

**Table:**

Contrast (for every sequence)							
Scan mode 1 = 2D 2 = 3D	Technique 1 = SE 2 = GE	Echoes	TE (msec) TR (msec)	Flip Angle	Half Scan	Number of signal acquisition	Total scan duration
